# Mitochondrial Dysfunction in Endothelial Cells: A Key Driver of Organ Disorders and Aging

**DOI:** 10.3390/antiox14040372

**Published:** 2025-03-21

**Authors:** Elena Grossini, Sakthipriyan Venkatesan, Mohammad Mostafa Ola Pour

**Affiliations:** Laboratory of Physiology, Department of Translational Medicine, Università del Piemonte Orientale, 28100 Novara, Italy; sakthipriyan.venkatesan@uniupo.it (S.V.); 20046522@studenti.uniupo.it (M.M.O.P.)

**Keywords:** ageing, endothelial cells, mitochondria, liver diseases, neurodegenerative disorders, renal diseases, reactive oxygen species

## Abstract

Mitochondria are of great importance in cell biology since they are major sites of adenosine triphosphate (ATP) production and are widely involved in different cellular pathways involved in the response to stress. During ATP production, reactive oxygen species (ROS) can be produced. While a small amount of ROS may be important for the regulation of physiological processes, at elevated levels they can turn into harmful agents leading to cellular damage. From a pathological perspective, it could be particularly interesting to focus on mitochondrial function in endothelial cells since they may be involved in the development of aging and in the onset of different diseases, including renal, cardio-metabolic, liver and neurodegenerative ones. However, to date, there are no surveys which address the above issues. To fill this gap, it may be valuable to collect recent findings about the role of mitochondria in the regulation of endothelial function, not only to increase knowledge about it but also for clinical applications. Here, we overview the most recent knowledge about the above issues in the view of characterizing the role of mitochondria in endothelial cells as an innovative potential target for the prevention of aging, as well as the treatment of the above pathological conditions.

## 1. Introduction

The endothelium acts as an important biological and mechanical barrier between the underlying tissue and circulating blood and facilitates nutrient transport and the elimination of waste products [1,2].

Considering all the above functions, it is quite obvious that endothelial dysfunction could act as an important player in the onset of many pathological conditions, such as cardiovascular, metabolic, renal, and neurodegenerative diseases, as well as aging. The mechanisms underlying those effects could be related to mitochondrial dysfunction [1,3].

Mitochondria are known as “power plants”, since they synthesize 90% of the ATP required for cell metabolism. Also, they are involved in the regulation of different features of cellular metabolism, such as ion homeostasis, cell growth, and signaling and redox balance, and for these reasons, they are an essential element in both cell survival and in mechanisms underlying cell death [4]. The proper mitochondrial function does strictly depend on mitochondrial membrane potential, which is generated by complexes I, III, and IV and provides the full oxidative phosphorylation (OxPhos) capacity to produce adenosine triphosphate (ATP) [5].

Also, regarding endothelial cells, mitochondria are of great importance since they are involved in the regulation of processes such as angiogenesis, apoptosis, and cellular aging [6]. For these reasons endothelial cell mitochondria play a pivotal role in the onset and evolution of the above-mentioned pathological conditions.

In detail, their involvement could be linked to the regulation of a series of events that ultimately lead to increased oxidative stress and programmed mechanisms, including apoptosis, necroptosis, pyroptosis, ferroptosis, and autophagy, as well as non-programmed necrosis [7].

For these reasons, targeting mitochondrial function could serve as a potential strategy for therapeutic interventions, offering new insights into treating those diseases.

While many studies have linked endothelial mitochondrial dysfunction to various clinical conditions, there is currently no comprehensive data that integrates those findings or clearly elucidates the role of mitochondrial endothelial cell dysfunction in the pathophysiology of neurodegenerative, renal, cardiovascular, and hepatic disorders, as well as in the onset of aging.

The aim of this review is to provide new insights into how mitochondrial dysfunction can compromise endothelial cell function, potentially leading to the onset of various organ disorders. To achieve this, we summarized the latest findings on the physiological and pathological mechanisms regulating mitochondrial function and their role in maintaining endothelial cell structure and integrity. Additionally, we highlighted the key intracellular pathways involved in these processes in order to increase the readers’ interest in the issues and contribute a novel perspective to the field.

Indeed, elucidating the intracellular mechanisms which regulate mitochondrial function in endothelial cells may also be valuable for highlighting the potential therapeutic applications of targeting mitochondria in endothelial cells in order to treat those pathological conditions.

As a whole, addressing the above-mentioned issues could pave the way for future perspectives for better understanding the pathophysiological basis of endothelial cell dysfunction-related diseases and improving their clinical management, particularly for conditions which remain challenging to treat.

## 2. Mitochondria’s Structure and Origin

### 2.1. Origin

According to the endosymbiotic hypothesis, the primitive mitochondria were engulfed by the ancestral anaerobic eukaryotic cells, though modern perspectives suggest an endosymbiotic event involving a non-eukaryotic archaeon, which then created a beneficial interaction for both organisms [8]. This interaction produced mutual benefits as the host cell provided a secure environment for bacterial growth, and mitochondria were able to significantly elevate the cellular energy production by synthesizing ATP via the respiratory chain [9].

### 2.2. Structure

**Figure 1:**
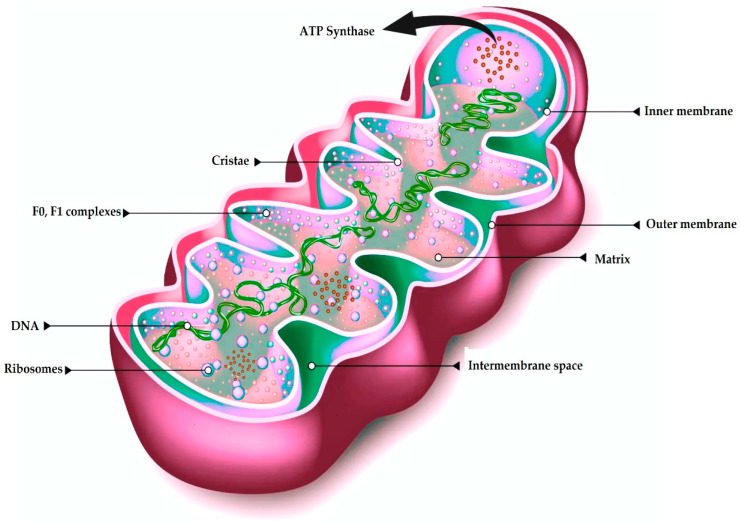
Mitochondria structure. DNA: deoxyribonucleic acid, ATP: adenosine triphosphate. Modified from https://centromedicomanzanera.com/it/lenergia-dellovulo-i-mitocondri (accessed on 11 February 2025).

Mitochondria are unique among organelles in having two membranes: an outer membrane (OM) and a morphologically complex inner membrane (IM) that subdivide the organelle into distinct compartments (Figure 1). The IM comprises cristae membranes (CMs), which protrude into the organelle’s interior, and the inner boundary membrane (IBM) facing the OM. These membranes define several aqueous compartments: the intermembrane space (IMS) between the OM and IBM, the intracristal space enclosed by the CM, and the matrix at the core of the organelle [7,10]. ATP synthase comprises two parts: F_1_ (with catalytic ATP synthesis sites) and F_o_ (a proton-driven turbine) whose coupled proton flow powers the rotor’s rotation and drives ATP production [11].

Notable structural features include crista junctions, which connect cristae to the IBM, and crista tips at the distal ends of cristae. Mitochondria typically appear as ovoid or tubular structures approximately 0.5–1 μm in width and 1–5 μm in length, although their critical ultrastructural components measure on the nanometer scale (e.g., membranes ~6–8 nm thick, cristae junction widths of 12–40 nm, cristae lumen ~25–30 nm) [12]. Because of the organelle’s endosymbiotic origin, the two membranes exhibit notable variations in lipid composition, permeability, structure, and the traits and functions of the transmembrane proteins [13].

## 3. Mitochondrial Genomic Structure

The mitochondrial deoxyribonucleic acid (DNA) (mtDNA) of most mammalian species encodes 13 highly hydrophobic polypeptides derived from 11 mitochondrial ribosomal ribonucleic acids (mRNAs), which are cotranslationally integrated into the IM, alongside various genes essential for the respiratory chain function. The mammalian mitochondrion comprises approximately 1200 proteins, while their genome is characterized by a compact, circular, double-stranded DNA structure. Moreover, mitochondrial DNA (mtDNA) encodes a full set of 22 mitochondria-specific transfer RNAs, facilitating protein synthesis through a modified genetic code [14]. Given the essential role of mitochondrial genes in ATP production, it is expected that the mitochondrial genome possesses high stability in its structure. However, mtDNA’s mutagenesis rate is significantly elevated in comparison to that of nuclear DNA [15].

## 4. ATP Generation in Mitochondria

### 4.1. Oxidative Phosphorylation System

**Figure 2:**
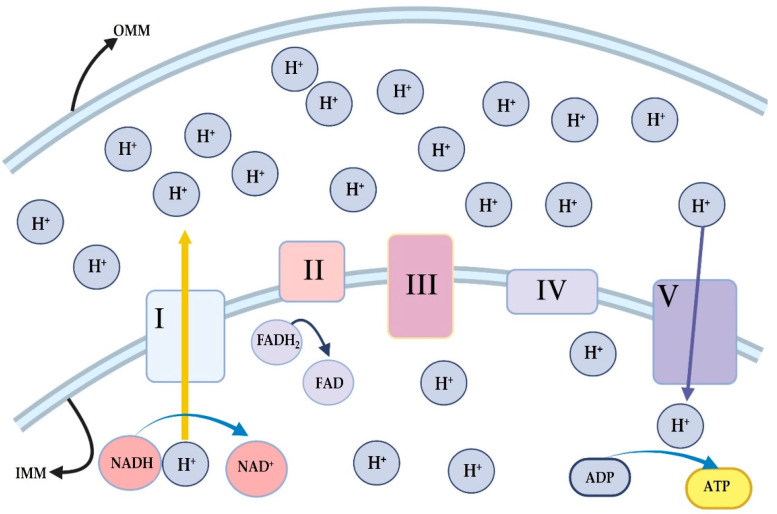
Mitochondria electron transport chain. I: complex 1, II: complex 2, III: complex 3, IV; complex 4, V: complex 5, IMM: inner mitochondrial membrane, OMM: outer mitochondrial membrane, FADH_2_: flavin adenine dinucleotide, FAD: flavin adenine dinucleotide, NADH: nicotinamide adenine dinucleotide, NAD^+^: nicotinamide adenine dinucleotide, ATP: adenosine triphosphate, ADP: adenosine diphosphate, and H^+^: hydrogen ion. Image was generated using biorender.com.

Oxidative phosphorylation (OxPhos) is the primary ATP-generating mechanism in mitochondria, where reducing equivalents [(nicotinamide adenine dinucleotide (NADH) and flavin adenine dinucleotide (FADH2)] from glycolysis and the tricarboxylic acid cycle fuel the electron transport chain (ETC) [16]. The ETC comprises four complexes (I–IV) that sequentially transfer electrons, creating a proton gradient across the inner mitochondrial membrane, which drives ATP synthesis by Complex V (ATP synthase) [17] (Figure 2). Accessory proteins modulate OxPhos, and glucose is the main cellular energy substrate, undergoing glycolysis in the cytoplasm to produce pyruvate, which is converted to acetyl-coenzyme A (acetyl CoA) for entry into the tricarboxylic acid cycle. In addition to generating ATP, the ETC also produces reactive oxygen species (ROS), primarily superoxide, which is normally neutralized by antioxidant systems. However, dysfunctional ETC activity can lead to excessive ROS production and attendant cellular damage [18].

### 4.2. Mitochondrial ROS

**Figure 3:**
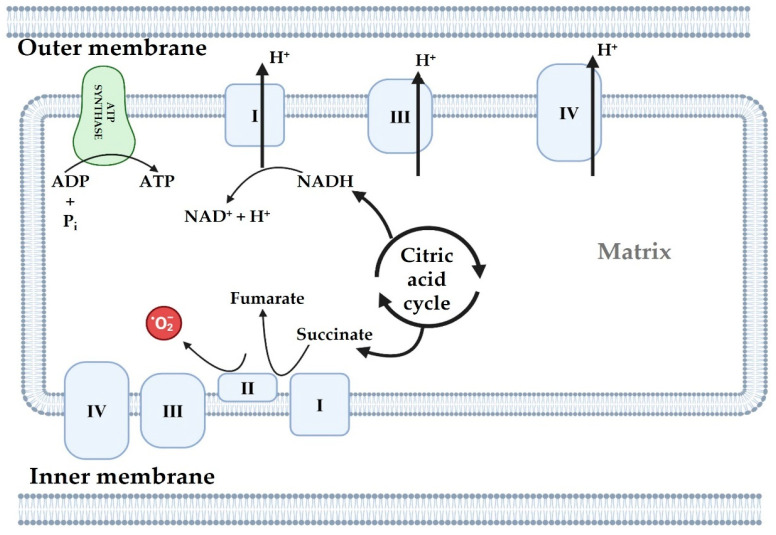
ROS production in mitochondria. ATP: adenosine triphosphate, ADP: adenosine diphosphate, P_i_: phosphate ion, **·**O_2_^−^: superoxide anion, NADH: nicotinamide adenine dinucleotide, NAD^+^: nicotinamide adenine dinucleotide, and H^+^: hydrogen ion. Figure was taken from Wikipedia.

Although complexes I and III are commonly recognized for their role in mitochondrial ROS (mtROS) production, complex II may also function as a source of ROS. As part of the Krebs cycle, complex II oxidizes succinate to fumarate and serves as the site for ubiquinone reduction in the ETC (Figure 3). It is important to note that, complex II produces superoxide anion (O_2_^−^), while the activity of complexes III and I is inhibited. The synthesis of ROS by complex II’s flavin site should proceed through a forward mechanism involving the release of electrons from succinate, or in a reverse mechanism where electrons are provided through a reduced ubiquinone pool [19].

## 5. Mitochondrial Dynamics

**Figure 4:**
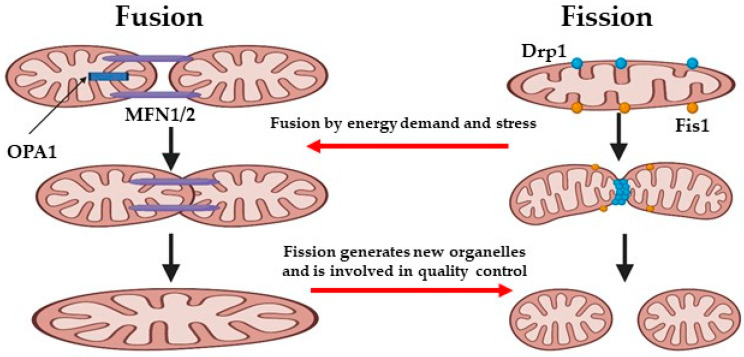
Mitochondrial dynamics. OPA1: optic atrophy protein 1, MFN 1/2: mitofusin 1/2, Drp1: dynamin-related protein, and Fis 1: mitochondrial fission 1 protein. Image was generated by using biorender.com.

Mitochondria continuously undergo fission, fusion, mitophagy, and transport cycles, which regulate their morphology, quality, quantity, and their distribution within cells, as well as their function [20]. Mitochondrial dynamics facilitate the removal of damaged components from the mitochondrion, or the elimination of dysfunctional mitochondria through mitophagy, to prevent further cellular damage [21]. The regulated balance of mitochondrial dynamics is a crucial element for the optimal function of mitochondria and determining cell fate (Figure 4) [22]. Concisely, fission contributes to mitochondrial quality control by the elimination of impaired or dysfunctional mitochondria and promotes apoptosis in the face of severe cellular stress. In contrast, fusion enables the mixing and exchange of the intramitochondrial contents between mitochondria, which helps sustain mitochondrial function [23]. Recent studies emphasized the crucial role of mitochondrial dynamics in preserving endothelial cell function and reducing oxidative stress. It is important to note that, therapeutic strategies aimed at regulating mitochondrial respiration and ROS levels, such as promoting OxPhos or mitochondrial fission suppression, were shown to be very promising for addressing endothelial cell damage caused by ischemia/reperfusion (IR) injury [5].

To form larger organelles, individual mitochondrial clusters are able to fuse, the size of which depends on the cell type, ranging from a few tens to several thousand [24]. These clusters connect dynamically to form an extensive reticular network, which can extend throughout the entire cell volume. The components of this network are cylindrical structures with diameters of a few hundred nanometers and are formed by the OM and IM of two separate mitochondrial clusters, resulting in the complete mixing of their contents, including their mtDNA, within 12 h. OM fusion is governed by the mitofusins, (Mfn)1 and Mfn2, and optic atrophy protein 1 (OPA1), whereas IM fusion is regulated by the mitochondrial fission 1 protein (FIS1) and Dynamin-related protein 1 (Drp1) [25].

Notably, OM fusion may occur independently from OxPhos, whereas IM fusion requires the enzymatic cleavage of OPA1, which is activated by high membrane potential, which implies that only healthy and active mitochondria can fuse properly [26]. Mitochondrial fission inhibition, obtained by downregulating FIS1 and Drp1, may ultimately lead to the inhibition of endothelial oxidative stress and to an improved endothelial function, by modulating endothelial nitric oxide synthase (eNOS) activation and nitric oxide (NO) bioavailability. The absence of additional benefits from an intracellular ROS scavenger indicates that elevated levels of mitochondrial segregation may increase ROS and disrupt endothelial function [27].

## 6. Mitochondria and Endothelial Cells Under Physiological Conditions

One of the main functions of endothelial cells is to isolate the blood from the arterial wall. In this way, they are involved in protecting the underlying tissue against the harmful effects of the bloodstream circulating factors [5]. In addition, to exert a role as an important barrier, the endothelial cells also play a role in the regulation of vascular tone through the release of vasodilators or vasoconstrictors and maintain blood vessels’ integrity by synthesizing extracellular matrix components [28]. Since endothelial cells are directly in contact with blood cells, they are recognized as “frontline” cells in fighting vascular diseases. The particular location of endothelial cells exposes them to both beneficial and detrimental molecules, such as inflammatory cytokines, ROS, oxygen, nutrients, hormones, etc. [29,30]. The presence of receptors on these cells enable them to detect changes in arterial blood pressure, blood flow, and the biochemical composition of the blood, and in case any adverse conditions are detected they can elicit a series of protective effects aimed at maintaining vascular homeostasis, thanks to the secretion of anti-inflammatory molecules, the regulation of coagulation, and the activation of repair mechanisms [31].

Furthermore, NO is one of the most critical components produced by endothelial cells, playing a key role in maintaining cardiovascular homeostasis and supporting a thrombolytic vascular environment [32]. The NOS produces NO through a redox reaction using L-arginine and molecular oxygen as substrates and reduced nicotinamide adenine dinucleotide phosphate and (6R-)5,6,7,8-tetrahydrobiopterin as cofactors [33].

Three well known isoforms of NOS are neuronal (n)NOS/NOS1, inducible (i)NOS/NOS2, and eNOS/NOS3 [34]. The eNOS generally is located in endothelial cells but it can be also found at lower levels in other cells, such as immune cells, platelets, and cardiomyocytes [33]. Due to the high reactivity and short-lived nature of NO, the activity of NOS is required to be strictly regulated. Of the three isoforms, only eNOS and nNOS are highly dependent on Ca^2+^ concentration for their activation [35].

When extracellular stimuli, such as vascular endothelial growth factor or shear stress, activate their respective receptors some intracellular pathways, such as those involving phospholipase C gamma 1 protein kinase B (Akt) and extracellular signal-regulated kinases 1/2, are activated and this, finally, leads to an increase in cytosolic Ca^2+^ levels, which allows Ca^2+^-calmodulin to bind to eNOS, enhancing its activation and the subsequent NO release. It should be highlighted that the activation of eNOS can also be related to the stimulation of cyclic adenosine monophosphate-dependent pathways involving protein kinase A and Akt, which drives eNOS phosphorylation, too [36,37].

In the vascular compartment, NO causes vasodilation by stimulating the enzyme soluble guanylate cyclase in smooth muscle cells. This leads to an increase in cyclic guanosine monophosphate levels, which lowers intracellular Ca^2+^ concentrations and promotes smooth muscle relaxation, ultimately causing vasodilation [38].

Differently from other cells, the endothelial ones have lower energy needs and produce ATP synthesis mainly by glycolysis. Furthermore, ROS production by endothelial mitochondria is known to play a significant role in regulating signaling cellular responses. In endothelial cells, mitochondria work like central oxygen sensors in the vascular system, acting as local environmental sensors [6]. Mitochondrial homeostasis and remodeling are further key features to maintain endothelial cell function by modulating ROS and calcium movements among other mechanisms [5]. Regarding this issue, it has been described that mitochondrial depolarization can increase NO production through a Ca^2+^-dependent eNOS activation and the involvement of intracellular signaling leading to Akt phosphorylation [39].

As observed in all cells, as well as in endothelial cells, mitochondria regulate ATP production through OxPhos, which supports vital cellular processes and vascular health. Also, the mitochondrial membrane potential, maintained by ETC complexes, is crucial for ATP synthesis, in endothelial cells as well. Mitochondria’s role in mediating ROS production, at moderate levels, may support signaling and vascular repair mechanisms. Indeed, mitochondrial ROS are tightly regulated products in endothelial cells and play pivotal roles in both physiological and pathophysiological settings, through the modulation of shear stress-induced vasodilation, hypoxia signaling, autophagy, and proinflammatory activation. Regarding vasodilation, it was shown that the shear stress-dependent NO release in endothelial cells could be related to the mitochondrial ROS release [40]. In particular, among various ROS, H_2_O_2_ could increase the Ca^2+^ concentration and regulate the eNOS activity [41]. However, excessive ROS, often due to mitochondrial dysfunction, can trigger endothelial activation, inflammation, and vascular diseases by affecting NO release too [42]. In brief, in pathological conditions oxidative stress can cause the eNOS uncoupling [43]. Uncoupled eNOS is involved in the production of superoxide anion instead of NO. In turn, superoxide anion can react with the small amount of NO, which is still produced by eNOS, to generate peroxynitrite anion, which is another kind of ROS. In mitochondria, peroxynitrite anion can overwhelm scavenging and repair systems and impair mitochondrial energy and membrane permeability, which contributes to mitochondrial dysfunction and ROS production [41].

It should also be noted that alterations in endothelial cell function can affect the activity of mitochondria by inducing changes in mitochondrial dynamics [26]. Regarding this issue, the inhibition of mitochondrial fission, obtained by blocking FIS1 and Drp1, was able to counteract endothelial oxidative stress and improve endothelial function, by changes in eNOS activation and in NO bioavailability. The lack of an additional benefit from an intracellular ROS scavenger suggested that enhanced mitochondrial segregation may increase ROS and damage endothelial function [27].

The metabolic flexibility of endothelial cells, adapting between oxidative phosphorylation and glycolysis depending on oxygen availability and shear stress, further underscores the dynamic interplay between mitochondria and vascular health [44].

## 7. Endothelial Cells and Mitochondria Dysfunction and Aging

Aging is hypothesized to be a degenerative phenomenon instigated by the accumulation of cellular damage, which subsequently culminates in cellular dysfunction, tissue failure, and eventual mortality [45]. During aging the functional activity of mitochondria progressively declines, which leads to an increased production of ROS that, in turn, impair mitochondrial function and furtherly potentiate the release of free radicals [6]. Recently, nine candidate hallmarks of mammalian aging have been described and subdivided into the primary hallmarks (genomic instability, telomere attrition, epigenetic modifications, and loss of proteostasis), the antagonistic hallmarks (mitochondrial dysfunction, deregulated nutrient sensing, and cellular senescence), and the integrative hallmarks (stem cell exhaustion and altered intercellular communication) [46]. These disruptions precipitate an augmented production of ROS, resulting in oxidative modifications and the accumulation of mutations within mtDNA, which substantially contribute to cellular dysfunction and the aging process. Mitochondrial dysfunction, which arises from both structural and functional alterations, is inherently associated with age-related degenerative pathologies [47]. The decline in mitochondrial functionality has been extensively documented to occur with advancing age, parallel to the emergence of morphological changes in mitochondria, such as the presence of abnormally rounded mitochondria in senescent mammals [45].

A disruption characterized by the excessive production of ROS in conjunction with a constrained cellular antioxidant defense mechanism precipitates oxidative stress. The involvement of ROS in the aging process has been substantiated through numerous studies that illustrate a progressive increase in ROS levels and oxidative damage concomitant with chronological aging. Moreover, ROS production is exacerbated by the inhibition of mitochondrial functionality; mitochondrial dysfunction escalates with advancing age, and several age-related pathologies are linked with heightened ROS production and oxidative stress [46]. In endothelial cells, this imbalance reduces NO bioavailability, impairing endothelial-dependent vasodilation and promoting arterial stiffness [19,48].

## 8. Endothelial Cells and Mitochondria in Cardio-Metabolic Dysfunction

That endothelial dysfunction represents a significant contributor for the onset of cardiac injury is widely accepted. Myocardial microvessels play a central role in maintaining vascular barrier function, vascular tone, and myocardial perfusion [49,50], thanks to autocrine, paracrine, and endocrine mechanisms, which involve the release of vasoactive agents and cell specific adhesion molecules. Also, in myocardial vessels, endothelial cells contribute to blood homeostasis and fluidity and to immune and inflammatory responses; it was reported that the endothelial damage may trigger apoptosis, resulting in a decline in capillary density within the heart as well. For all the above reasons, all conditions characterized by endothelial dysfunction and myocardial microvascular injury could lead to the onset of cardiovascular diseases (CVD) [51,52].

As discussed above, it is important to note that changes in cardiac endothelial function have been recognized as being closely related to mitochondrial abnormalities, which, in turn, may regulate the structure and function of endothelial cells themselves [1]. Mitochondria, which, as described above, are considered as the powerhouses of the cell, are not only involved in ATP production but also in the regulation of key biological processes in vascular endothelial cells, such as redox signaling, aging, angiogenesis, and inflammation [6]. For those reasons, various chronic conditions, like those characterized by hypoxic-ischemic injuries, atherosclerosis, stroke, diabetes, and hypertension, can be significantly impacted by mitochondrial dysfunction [53].

Among various mechanisms playing important roles in maintaining the functionality of mitochondria in vascular endothelial cells, there are those involving the sirtuin (SIRT) family, ionic homeostasis, and hormones. Regarding sirtuins, SIRT 1 and SIRT3 were reported to exert cardioprotective effects by modulating mitochondrial biogenesis and genes. Also, they can prevent IR injuries through a pathway related to the adenosine 5′-monophosphate (AMP)-activated protein kinase (AMPK)/SIRT1/peroxisome proliferator-activated receptor γ coactivator 1-alpha (PGC-1α), which is involved in counteracting mitochondrial oxidative stress damage [54].

Also, changes in ionic homeostasis could induce endothelial dysfunction and cardiomyocyte apoptosis through perturbations in mitochondria. It is well known that mitochondria can accumulate Ca^2+^ which triggers a phenomenon called mitochondrial permeability transition pore opening (mPTP), characterized by the opening of high-conductance pores in the IMM. This process is followed by mitochondrial swelling and the rupture of the mitochondrial membrane and leads to the release of mitochondrial proteins, including cytochrome c, into the cytosol [1].

In addition to Ca^2+^ calcium overload, iron overload can also lead to mitochondrial dysfunction in endothelial cells. It was shown that an increase in iron in the cytoplasm may generate an overload of ROS, by inhibiting dimethylarginine dimethylaminohydrolase and accumulating asymmetric dimethylarginine (ADMA). ADMA, in turn, would reduce the NO release by the inhibition of eNOS and the activation of eNOS uncoupling. In addition, excessive ROS may weaken the mitochondrial membrane potential and cause the mPTP. All the above events would generate more ROS and establish a vicious cycle potentiating ROS production [55].

Another important issue, which could represent a pathophysiological mechanism at the root of the onset of CVD, is that mitochondria could act as mechanosensitive organelles and participate in the onset of cardiac failure through their capability to perceive mechanical stress generated by cardiac contraction and relaxation, arterial blood pressure, and blood flow. In cardiovascular cells, it is well known that physiological mechanical stress, mainly shear stress and cyclic stretch, are able to preserve mitochondrial homeostasis, whereas in pathological conditions, they may impair ATP production and the ETC, leading to abnormal Ca^2+^ transit and ROS accumulation [56].

One of the most frequent causes of CVD, in which damage to the microcirculation and endothelial dysfunction play a crucial role, is diabetes. Also, the loss of microvascular integrity and barrier function is considered as the first step in diabetic vascular complications [6].

In the presence of microvascular endothelial dysfunction, the impairment of blood flow regulation would prevent it from adjusting to the increased myocardial oxygen requests from rest to stress conditions [57]. This imbalance may lead to mitochondrial dysfunction, which would induce oxidative stress, activate endothelial cell apoptosis, and exacerbate the progression of diabetic microvascular complications.

It should be highlighted that, in the presence of diabetes, endothelial damage can also be derived from a primary alteration of mitochondrial function caused by changes in blood glucose levels. It was found that high glucose exposure can lead to increased fission and reduced fusion, resulting in mitochondrial fragmentation. As a consequence, the impairment of mitochondrial metabolism, inhibition of ATP synthesis, and activation of mitochondrial-dependent apoptosis could arise [58].

Furthermore, glycated or oxidized low-density lipoprotein (LDL), which can be found in diabetic conditions, could hamper mitochondrial respiratory function through the reduction in mitochondrial oxygen consumption and the membrane potential and the impairment of the activity of ETC complex enzymes [59,60]. Also, glycated or oxidized LDL could activate mitochondrial oxidative stress in endothelial cells by increasing nicotinamide adenine dinucleotide phosphate hydrogen oxidase [7].

In diabetic patients, the mitochondrial quality surveillance system can address the endothelial stress by changing the mitochondrial metabolism, dynamics, mitophagy, and biogenesis. For these reasons, targeting mitochondrial dysfunction may represent a therapeutic strategy for the management of CVD in diabetic patients.

Indeed, the use of metformin or 5-aminoimidazole-4-carboxamide ribonucleoside or antioxidant strategies can be useful for maintaining the cardiac microvascular integrity by enhancing mitochondrial biogenesis, inhibiting mitochondrial fission, and increasing mitochondrial fusion and mitochondria proliferation. Pathways related to AMPK and PGC1α would be involved in those effects [6].

Also, the therapeutic drug empagliflozin could maintain cardiac microvascular endothelial cells in diabetic patients by inhibiting mitochondrial fission through AMPK-dependent pathways [61].

## 9. Mitochondrial Dysfunction and Renal Diseases

The kidney is recognized as one of the most metabolically demanding tissues within the human anatomical framework, with both the kidney and the heart exhibiting elevated resting metabolic rates relative to the brain and other physiological structures. The kidney necessitates the second highest mitochondrial density and oxygen utilization, following the heart, in order to facilitate a multitude of cellular functions and processes, which encompass the elimination of metabolic waste, nutrient reabsorption, homeostatic fluid balance, and blood pressure regulation [62,63,64].

Contemporary research endeavors have concentrated on elucidating the role of mitochondrial dysfunction in the etiology of various renal pathologies, notably acute kidney injury (AKI) and chronic kidney disease (CKD). A correlation has been established between AKI and an augmented susceptibility to the onset of CKD. Both AKI and CKD manifest as extensive clinical syndromes with significant implications for morbidity and mortality, encompassing diverse etiological factors and presenting considerable challenges to global public health initiatives [65]. The presence of mitochondrial dysfunction is associated with diminished ATP levels and elevated ROS levels, culminating in renal impairment [62].

Mitochondria have been acknowledged as pivotal components in AKI, exhibiting dual functionalities as the primary energy source for cells and as crucial regulators of cellular apoptosis. Initial observations have indicated alterations in mitochondrial morphology during AKI. Ischemia, a principal causative factor of AKI, leads to a reduction in mitochondrial quantity and induces structural changes, characterized typically by swelling and the loss of intermitochondrial cristae, as a consequence of ATP depletion and diminished membrane potential [65].

The main features of CKD are inflammation and fibrosis and are involved in the transition from AKI to CKD. In particular, necrotic cells release damage-associated molecular patterns (DAMPs), which activate similar pattern recognition receptors on tissue-resident cells (such as dendritic cells) or attract leukocytes, prompting these cells to secrete pro-inflammatory cytokines and chemokines. In both in vitro and in vivo models of acute tubular injury, it was found that the release of DAMPs and the activation of nucleotide-binding oligomerization domain-like receptors could induce a persistent renal injury and facilitate the transition from AKI to CKD [66].

## 10. Neurodegenerative Diseases

With the increase in research in the field, the knowledge relating to the role of mitochondria in the onset of neurodegenerative diseases has been improved. Busija DW et al. found that mitochondrial function remains elevated for up to 48 h in rat brain vessels after transient middle cerebral artery occlusion. This observation highlights the central role of endothelial cell mitochondria in the recovery of vascular function post-stroke and may provide new perspectives for the therapeutic treatment of stroke [67].

Also, Deng et al. found that the use of the δ-opioid receptor agonist, [D-ala2, D-leu5]-Enkephalin (DADLE), could significantly activate mitochondrial autophagy in brain microvascular endothelial cells by upregulating the expression of the transient receptor potential vanilloid subtype 4 (TRPV4). In this way, they observed a reduction in the cell damage caused by I/R injuries. The mechanisms through which the activation of TRPV4 could promote vascular regeneration and help the recovery of ischemic stroke patients have been shown in previous studies. In addition, it was reported that the protective effects of DADLE could be blocked by TRPV4 inhibitors or RNA interference, which highlighted the pivotal role of TRPV4 in DADLE-induced mitochondrial autophagy. Finally, it was found that DADLE could exert protective effects through the activation of mitochondria autophagy, not only by upregulating the TRPV4 expression but also by increasing calcium ion influx [6].

### 10.1. Blood Brain Barrier, Mitochondria, and Endothelial Cells

The prevalence of central nervous system (CNS) diseases is considerably higher than that of other systems. Despite the great research progress on this issue, the development of therapeutic strategies for the treatment of neurodegenerative diseases is unfortunately slowed down by many issues, including significantly longer drug development times and lower success rates than other systemic drugs. These difficulties are related to the complex and poorly understood pathogenesis of brain diseases, with particular reference to the role of the blood–brain barrier (BBB) [68,69].

Regarding this issue, in recent years, many studies have characterized the BBB as an important player in a huge number of CNS diseases, since it is essential for maintaining a relatively stable physiological condition in the brain. In the case of pathology, BBB alterations allow the accumulation of toxic substances and inflammatory cells around blood vessels and their entry into the CNS [70]. In addition, the pathological mechanisms at the root of BBB disruption would involve brain endothelial cell dysfunction, too [71]. Brain endothelial cells are highly susceptible to oxidative stress, and the mitochondrial respiratory chain generates over 90% of intracellular ROS. Thus, maintaining the BBB’s integrity requires the strict regulation of mitochondrial respiration. Brain endothelial cells are located between the CNS and the circulatory system, and the mitochondria of brain endothelial cells may have a pivotal role in the onset of CNS diseases [72]. Many studies have demonstrated that the high oxidative stress state of brain endothelial cells is closely related to the excessive mtROS production [73].

As a consequence of all the above mechanisms, inflammatory responses, impaired angiogenesis, and reduced local cerebral blood flow would arise as a consequence of BBB damage and could represent the pathophysiological condition at the root of neurodegenerative diseases, such as Alzheimer’s disease, Parkinson’s disease, ischemic stroke, and amyotrophic lateral sclerosis.

Since there is an urgent need to identify new therapeutic targets to treat CNS diseases, increasing knowledge about the role of mitochondria could represent a useful additional weapon.

### 10.2. Alzheimer’s Disease (AD)

Since AD is a progressive neurodegenerative disorder, cerebrovascular dysfunction is one of the earliest events, as well as for mixed dementia [6].

Mitochondrial dysfunction in endothelial cells is a key factor in the pathogenesis of AD. Gjumrakch Aliev et al. reported that increased ROS levels could damage cerebral vascular endothelial cells and impair both the integrity and function of the BBB. Specifically, in oxidative stress conditions, the ROS-induced NO degradation would impair the vasodilatory effect of NO, while enhancing endothelin-1-mediated vasoconstriction, which, in turn, could lead to chronic cerebral hypoperfusion and AD progression. Additionally, the deposition of Amyloid β protein (Aβ) around cerebral blood vessels may further amplify the vascular damage caused by ROS. In that study, they also showed a link between mitochondrial DNA depletion and oxidative damage, which is particularly evident in damaged neurons of AD patients.

In addition to AD, the loss of mitochondrial endothelial cell function plays a crucial role in the pathogenesis of cerebral amyloid angiopathy, by affecting energy metabolism and the production of ROS, NO, and reactive nitrogen species, as well as through changes in calcium signaling and the activation of apoptosis. It was shown that Aβ may exert detrimental effects on mitochondrial function in brain endothelial cells, leading to reduced ATP production and increased oxidative stress, ultimately impairing neurovascular function [74].

Also, Lyros et al. highlight the pivotal role of mitochondria in AD. Indeed, they observed their dual function as energy production hubs and key regulators of the brain’s microenvironment homeostasis. Since Aβ deposition could trigger mitochondrial dysfunction and activate programmed cell death in endothelial cells, Aβ may act as an upstream event leading to endothelial dysfunction in AD. All the above processes could activate a vicious cycle in which mitochondrial dysfunction could increase ROS levels, which, in turn, would enhance Aβ production [1]. Further investigations suggested that AD may disrupt both mitochondrial biogenesis and the dynamics of mitochondrial fission and fusion, thereby leading to an unequal distribution of mitochondria within neuronal cells [75]. Finally, the concomitant presence of mitochondrial DNA loss and of markers of oxidative damage in AD further support the pivotal role of mitochondria in the onset of neurodegenerative diseases through enhanced vascular dysfunction [76].

### 10.3. Huntington’s Disease (HD)

HD is a fatal, autosomal dominant neurodegenerative disorder characterized by limb tremors, cognitive decline, and behavioral disturbances. Pathological changes primarily affect brain regions such as basal nuclei (caudate nucleus, putamen, and subthalamic nucleus) and cerebral cortex, along with the hypothalamus. HD arises from mutations in the huntingtin gene, involving expanded cytosine-adenine-guanine repeats in exon 1. This leads to the selective degeneration of gamma-aminobutyric acid-ergic medium spiny neurons in the striatum. Furthermore, the misfolded huntingtin gene disrupts crucial processes, including axonal transport, signal transduction, and autophagy [77].

In the onset of HD, oxidative stress, calcium dysregulation, and impaired respiratory chain enzyme activity are widely considered as significant contributors. In mitochondria, increased fragmentation, reduced motility, and diminished respiratory function have been observed in both patients and experimental models. Additionally, it was found that the inhibition of the citric acid cycle, through the use of 3-Nitropropionic acid, could induce striatal degeneration resembling HD progression. It was also reported that glyceraldehyde-3-phosphate dehydrogenase, a key enzyme in glycolysis which in physiologic conditions would support mitochondrial activity via autophagy, in pathological conditions, such as those that can be found in HD, would lose this function as a consequence of its interaction with the mutant huntingtin gene [78,79,80].

### 10.4. Parkinson’s Disease (PD)

PD is a neurodegenerative disorder characterized by tremors, tonus, bradykinesia, and postural instability. It has been recently reported that oxidative stress, dysregulation of mitochondrial homeostasis, and ROS imbalance could be considered as key pathogenesis factors at the root of degenerative neuronal death in the midbrain substantia nigra, decreased striatal dopamine levels, and the accumulation of misfolded intracellular α-syn. In turn, co-localization of α-syn monomer with cardiolipin of the OM would promote the mPTP, increase ROS release, and cause oxidative stress and apoptosis in dopaminergic neurons. Also, changes in mitochondrial fusion and fission and the activation of mitophagy could represent important mechanisms for the onset of PD [81].

It is important to note that, not only the loss of function of dopaminergic neurons but also vascular alterations could play a role in the pathophysiology of PD. In particular, the activation of pericytes, BBB disruption, abnormal angiogenesis, and vascular regression have been reported. Also, early brain endothelial cell proliferation and dysfunction, which could be associated to α-syn deposition, were suggested to take part in the progression of PD. As for other neurodegenerative diseases, increases in BBB permeability could lead to a loss of function of the neurovascular unit (NVU) and induce neuroinflammatory responses and neuronal loss [82,83].

### 10.5. Amyotrophic Lateral Sclerosis (ALS)

ALS is a neurodegenerative disease, whose underlying mechanism has not yet been clarified, characterized by a progressive deterioration of upper and lower motor neurons. Various targets, such as neurotoxic microglia, astrocytic excitotoxicity, impaired DNA repair, protein misfolding/aggregation, impaired proteolysis, mitochondrial dysfunction, and axonal transport defects, could be involved in the ALS pathogenesis. Although in about 10–20% of ALS cases there is a genetic basis, environmental factors could also account for its onset. For instance, age, male sex, smoking, exposure to β-N-methylamino-L-alanine, physical activity, trauma, and agricultural chemicals have been widely reported as factors playing a role in developing ALS, [84,85,86,87]. Recently, changes in the NVU, which is a network composed of vascular cells, glial cells, and neurons, and in mitochondria function have been hypothesized to be involved as well. In particular, the disruption of the NVU could compromise the BBB or blood–spinal cord barrier, allowing harmful substances to enter into the central nervous system and causing damage to motor neurons. Also, recent evidence has revealed an imbalance of oxidants and antioxidants in the plasma of ALS patients. Furthermore, ALS patient plasma has been shown to negatively affect the survival of vascular endothelial cells and astrocytes, as well as their mitochondrial function and levels of oxidative stress [84,88].

### 10.6. Plant Antioxidant Supplementation for Improving Mitochondrial Function in Neurodegenerative Disorders

Starting from the assumption that alterations in mitochondrial function and oxidative stress may be at the basis of the onset of neurodegenerative diseases, it is quite obvious how the use of antioxidants can have a therapeutic role in dealing with the aforementioned pathological conditions [89].

Antioxidants can be found in many beverages, fruits, and vegetables, and they can be synthesized in laboratories; since they can counteract cellular oxidative damage they can have several benefits for the prevention/treatment of diseases, either used alone or in combination with other medications [90].

Among various antioxidants, the plants-derived ones have attracted significant attention in this field. It is important to note that clinical data highlighted the potential of polyphenol-rich diets to decrease the risk and alleviate symptoms of neurodegenerative disorders and to provide benefits for maintaining cognitive functions [91,92]. Due to their anti-inflammatory, anti-amyloid, and anti-oxidant properties, the polyphenols, in particular, could contribute to mitigating the progression of conditions such as AD, PD, HD, and ALS. In AD, green tea and white tea extracts have been used as therapeutic tool thanks to their role in counteracting the Aβ-mediated cytotoxicity. Also resveratrol, curcumin, quercetin, epigallocatechin gallate, genistein, naringenin, catechin, and rutin, which are main polyphenols contained in red wines, grapes, citrus fruits, soybean, cocoa, and berries, would exert their beneficial effects in the above conditions by inhibiting Aβ and oxidative stress and by maintaining the mitochondrial function [93,94,95,96,97]. Their therapeutic potential would be linked to the regulation of signaling pathways, such as Akt, nuclear factor erythroid 2-related factor 2, Janus kinase/signal transducer, the activator of transcription, nucleotide-binding and oligomerization domain-like receptor family pyrin domain-containing 3 inflammasome, and mitogen-activated protein kinase (MAPK), which play critical roles in neuroprotection and the body’s immune response [91,92].

It is also important to note that some of those polyphenols could exert protection in neurodegenerative diseases by modulating copper and zinc metabolism. These metals play a pivotal role in various biological processes, including energy production, antioxidant defense, ROS production, mitochondrial function, and neurotransmission. There is lots of evidence suggesting that alterations in copper and zinc homeostasis would contribute to the onset of age-related neurodegenerative diseases, such as AD, PD, HD, and ALS. For these reasons, the use of Metal–Protein Attenuating Compounds, copper/zinc chelators, and copper supplements has been suggested as a possible therapeutic tool for the treatment of the above conditions. In this context, natural compounds, like quercetin, luteolin, apigenin, resveratrol, curcumin, rutin, apigenin, and epigallocatechin gallate, were reported to be able to modulate plasma copper and zinc levels and counteract oxidative stress, neuroinflammation, and mitochondrial dysfunction [98,99].

Furthermore, it is important to note that, in addition to copper and zinc, iron accumulation may contribute to the onset of AD, through ferroptosis. In this process, lipid peroxidation and the depletion of antioxidants, such as glutathione, may lead to oxidative cell death. Thus, the use of agents able to chelate iron may be helpful for the treatment of AD. Regarding this issue, it was found that the flavonoids, like aspalathin, nothofagin, and quercetin, contained in Rooibos could act as radical scavengers, inhibit lipid peroxidation, and delay neurodegeneration by chelating iron [100].

In Table 1 data about the involvement of endothelial cells and mitochondria in health and diseases are summarized.

## 11. Mitochondrial Dysfunction and Liver Diseases

The liver serves as the body’s primary metabolic organ and boasts a remarkable capacity for regeneration and self-repair. Each hepatocyte houses between 1000 and 2000 mitochondria. However, defects or diminished function in these organelles can reduce ATP production, disrupt immune regulation, initiate programmed cell death, and slow liver recovery following injury. In particular, mitochondrial dysfunction, including loss of mitochondrial membrane potential, lowered respiratory chain complex activity, and decreased ATP generation, has been linked to the onset of liver fibrosis, cirrhosis, and cancer. Furthermore, a recent study revealed that markers of hepatic mitochondrial biogenesis, autophagy, and fission/fusion decline significantly as non-alcoholic fatty liver disease (NAFLD) progresses. Consequently, investigating the role of mitochondrial dysfunction in the development of liver diseases holds considerable importance [101,102].

Chronic hepatitis B virus (HBV) infection exerts its long-term pathogenic effects primarily through mitochondrial dysfunction and morphological alterations. Transmission electron microscopy reveals that HBV-infected hepatocytes undergo profound changes, such as disrupted cytoskeletons and swollen mitochondria lacking normal the tubular shape and cristae. Furthermore, both HBV and hepatitis C virus replication induce Drp1 expression and Parkin translocation, promoting mitochondrial fission and mitophagy. This shift in mitochondrial dynamics helps viruses evade innate immune responses, thus facilitating viral replication and enabling persistent infection [103,104,105].

In alcoholic liver disease, alterations in mitochondrial division also play a crucial role. Alcohol consumption spurs the formation of enlarged mitochondria, known as megamitochondria, which are often observed in liver biopsies. The key mediator of mitochondrial structural changes is Drp-1, a protein that drives organelle fragmentation. While Drp-1 inactivation exacerbates megamitochondria formation and reduces alcohol-induced damage in some contexts, recent research indicates that the inability to clear oversized megamitochondria through mitophagy leads to the accumulation of dysfunctional mtDNA and increased inflammation. Therefore, preserving the equilibrium between mitochondrial fission and fusion is of great importance for normal cellular function and immune regulation [43,106,107].

Similar mitochondrial dynamic imbalances underlie NAFLD or metabolic-associated fatty liver disease (MAFLD). In the pathogenesis of MAFLD, which is a complex and multifactorial condition, genetic predisposition, environmental factors, and metabolic dysregulation react with each other. In particular, the excessive intake of saturated fats, refined carbohydrates, and fructose has been associated with an increased risk of MAFLD and of its evolution towards metabolic-associated steatohepatitis (MASH), by accumulating fatty acids and contributing to the development of inflammation and lipotoxicity, insulin resistance, mitochondrial dysfunction, and oxidative stress. Alterations in mitochondrial function and the increased ROS release would lead to cellular damage and the activation of Kupffer cells and inflammatory pathways, which could, in turn, exacerbate mitochondrial dysfunction and perpetuate the damage [108].

Also, diets high in fat or cholesterol could provoke mitochondrial structural defects, manifesting as cristae loss and reduced levels of fusion proteins (OPA-1 and MFN1/2), coupled with increased Drp-1 expression. The early suppression of hepatocyte Drp1 can protect against simple steatosis by elevating proton leakage, which both enhances lipid oxidation and lowers ROS production. However, the later-stage knockdown of Drp1 may worsen non-alcoholic steatohepatitis (NASH) due to excessive lipotoxicity and a heightened mitochondrial stress response. Together, these findings underscore that striking an appropriate balance between mitochondrial fission and fusion is vital to averting worsening liver damage [109,110].

Since MAFLD often coexists with diabetes mellitus, and the two clinical conditions share the same pathogenesis, some clinical trials have evaluated the effectiveness of the use of anti-diabetic drugs for MAFLD treatment. Among various agents, metformin, pioglitazone, glucagon-like peptide-1 (GLP-1) agonists, and GLP-1/ gastric inhibitory polypeptide (GIP) coagonists have been reported to be effective as therapeutic tools in MAFLD.

The GLP-1 analogs liraglutide and semaglutide have been proven to delay the development of MASH through the modulation of mitochondrial function. Semaglutide can counteract mitochondrial dysfunction by enhancing autophagy and potentiate the response to oxidative stress in neurodegenerative diseases. In addition, semaglutide can bind with GLP-1 receptor (GLP-1R) to reduce peroxidation and improve the glucose–lipid metabolism in hepatocytes of MASH patients. It was reported that liraglutide could inhibit the development of MAFLD by increasing the levels of mitochondria-related structural proteins involved in fusion and fission, like Drp1, OPA1, and mitochondrial uncoupling protein 2, enhancing mitochondrial remodeling and restoring the expression of autophagic proteins, like Beclin 1, LC3, and SIRT1. When using the GLP-1 agonists with other drugs, such as the farnesoid X receptor agonists and the acetyl-CoA carboxylase inhibitor, stronger effects on transaminases and fat content can be observed than those found when using one drug [111].

It should be noted that, although GLP-1 agonists are very promising as therapeutic agents at the beginning of the disease, metabolic liver damage could recur after the end of the monotherapy. For this reason, the use of the dual GLP-1 and GIP receptor agonist has been approved by the Food and Drug Administration for the treatment of diabetes type 2. Indeed, the treatment of patients with MAFLD with dual incretin analogs was found to reduce hepatic steatosis and inflammation, enhance hepatic lipid metabolism and systemic insulin sensitivity, and counteract liver fibrosis [112,113].

## 12. Mitochondria as a Potential Therapeutic Target in Diseases

The majority of mitochondrial therapies are designed to either augment mitochondrial function or address the repercussions of mitochondrial dysfunction. Certain therapeutic approaches focus on enhancing the substrate availability for the respiratory chain, promoting electron transfer within the respiratory chain, or attempting to create a biochemical bypass of specific respiratory chain complexes. Additional strategies aim to mitigate the accumulation of toxic metabolites, augment ATP storage, or induce adaptations in mitochondria that enhance oxidative capacity [114]. The therapeutic strategies encompass symptomatic interventions, primarily including dietary modifications, physical exercise, exposure to hypoxia, and pharmacological treatments, which are predicated on pharmacological agents designed to (i) promote mitochondrial biogenesis; (ii) activate the NOS pathway; (iii) increase ATP production; (iv) bolster antioxidant defenses; (v) enhance the mitochondrial quality control mechanisms by stimulating dynamics (fission/fusion processes) and the autophagic degradation of impaired mitochondria; and (vi) target cardiolipin [115].

Mitochondrial ROS play a crucial role in both cellular signaling and endothelial cell dysfunction in the vascular system. Thus, therapeutic strategies that restore ROS levels to normal could be highly beneficial [116]. While clinical trials using non-targeted antioxidants (e.g., vitamins A and E, selenium, or β-carotene) have generally been disappointing, antioxidants specifically designed to target mitochondria may offer better outcomes [53].

### 12.1. Exposure to Hypoxia

The hypoxic response, which is a cellular mechanism facilitating adaptation in conditions of oxygen deprivation, was recognized in 2016 as a significant inhibitor of mitochondrial dysfunction and subsequently proposed as a viable therapeutic intervention for mitochondrial disorders [84]. A comprehensive genome-wide screen utilizing the Clustered Regularly Interspaced Short Palindromic Repeats (CRISPR)-associated protein 9 (Cas9) to induce complex III deficiency pharmacologically revealed that the inhibition of the Von Hippel–Lindau factor emerged as the most effective suppressor of mitochondrial dysfunction. Since the Von Hippel–Lindau factor may exert a negative regulatory influence on hypoxia-inducible factors (HIFs), its downregulation could lead to the activation of the HIF transcriptional response, which, in part, may redirect the cellular bioenergetic reliance towards mitochondrial OxPhos. In the zebrafish models, both the genetic and pharmacological activation of the HIF signaling pathway, as well as the hypoxic treatment in the knockout mouse model of Leigh syndrome, further substantiated the efficacy of this therapeutic paradigm [117].

### 12.2. Mitochondrial Function Targeted Drugs

Recent research highlights the importance of mitochondrial dynamics, encompassing fission, fusion, biogenesis, and mitophagy, in devising new therapeutic strategies for a variety of diseases [118]. By targeting specific proteins and signaling pathways involved in these processes, researchers aim to restore or enhance mitochondrial function. Mitochondrial Division Inhibitor 1 was among the first compounds to selectively inhibit Drp1, a key mediator of mitochondrial fission. Other fission-modulating agents, like Dynasore and P110, operate via similar mechanisms, collectively underscoring the potential of modulating the fission/fusion balance to protect against conditions such as neurodegenerative disorders [119].

Conversely, enhancing mitochondrial fusion can provide therapeutic benefits by preserving organelle integrity and function. Several interventions, including heme oxygenase-1 overexpression, melatonin treatment, and the compound hydrazone M1, boost MFN expression or activity, thereby supporting healthier mitochondrial networks. Mitophagy, a key mechanism in mitochondrial quality control, is regulated by pathways such as Phosphatase and tensin homolog deleted on chromosome 10-induced kinase 1 (PINK1) and Parkin RBR E3 ubiquitin-protein ligase. Compounds that activate this cascade, like kinetin, kaempferol, rhapontigenin, and SR3677, promote the selective removal of damaged mitochondria and are of great interest for diseases characterized by impaired mitochondrial turnover [10].

Beyond morphological regulation, considerable attention has been devoted to drugs targeting mitochondrial function and metabolism. For instance, cyclosporin A inhibits the mitochondrial permeability transition pore, Ru360 and MCU-i4 reduce mitochondrial calcium overload, and coenzyme Q both participates in electron transport and acts as a potent antioxidant. Idebenone, a coenzyme Q analog, demonstrates a favorable pharmacokinetic profile and shows promise for conditions like Leber’s hereditary optic neuropathy. Additional metabolic modulators include 2,4-dinitrophenol and its safer derivatives, which uncouple oxidative phosphorylation, as well as carnitine, which is vital for fatty acid transport into mitochondria. Meanwhile, resveratrol and 5-Aminoimidazole-4-carboxamide ribonucleotide enhance mitochondrial biogenesis and respiratory chain activity, in part by activating SIRT1 and AMPK pathways [120].

### 12.3. Gene Therapy

Despite significant advancements in oncology and some immune disorders, the use of cellular therapy for mitochondrial-associated diseases is still developing [121]. Furthermore, pioneering cellular therapeutic strategies, including gene therapy, liver transplantation, and mitochondrial transplantation, have been instrumental in enhancing mitochondrial quality for the prevention and treatment of diverse major diseases [122].

Mitochondrial replacement technologies offer a promising avenue for preventing the transmission of pathogenic mtDNA. In nonhuman primates, this approach has been achieved by transferring the spindle chromosomal complex from a donor oocyte into an enucleated egg harboring a healthy mitochondrion, producing embryos and viable offspring with nuclear DNA from the spindle donor and mtDNA from the cytoplast donor [123].

Gene therapy further expands therapeutic options for mitochondrial disorders, targeting either mutated nuclear DNA or mtDNA. Adeno-associated viruses (AAVs) are a particularly attractive vector due to their lack of pathogenicity and ability to remain in cells as episomes, minimizing insertional mutagenesis. Through various preclinical studies, mutated genes have been replaced or corrected using both AAV delivery and CRISPR/Cas9 tools [124].

Several mouse models illustrate the potential of AAV-mediated interventions. Early studies involved injecting AAV2 and encoding *ANT1* into Ant1 mice, which reversed mitochondrial myopathy by restoring normal adenosine diphosphate/ATP transport in muscle cells. Additional work has demonstrated the benefits of AAV-based therapies in conditions mimicking severe mtDNA depletion syndromes and Leigh syndrome. One challenge in translating these strategies lies in accurately replicating human mitochondrial pathophysiology in animal models. For example, *Mpv17* knockout mice do not naturally develop severe hepatic dysfunction typical of human MPV17-related disease. Researchers overcame this limitation by administering a ketogenic diet, which induced hepatic cirrhosis and liver failure in the mice. Treating these mice with AAV2/8 carrying a functional *MPV17* gene restored mtDNA content and OxPhos function, preventing liver disease progression. Overall, advancements in mitochondrial replacement, targeted gene therapy vectors, and innovative animal models underscore a rapidly evolving field. Although hurdles remain, particularly in replicating human disease phenotypes, these approaches collectively pave the way toward effective treatments or preventions for mitochondrial disorders [117,125,126,127,128].

### 12.4. Mitochondrial Transplantation

Mitochondrial transplantation constitutes a pioneering cellular therapeutic modality aimed at addressing significant pathologies, which encompasses the extraction and subsequent transplantation of healthy, functionally proficient mitochondria into defective cellular structures to supplant compromised organelles. Mitochondria transplantation can be performed using either autologous or heterologous approaches [129]. Various viable cells and tissues, including stem cells and skeletal muscle cells, can serve as sources for mitochondrial isolation and purification. This process can be performed using commercial kits or manual differential centrifugation methods [129,130]. Delivery methods can include direct microinjection, cell-mediated transfer utilizing Tunneling nanotubes, vesicle-mediated delivery and systemic delivery where mitochondria are conjugated to a carrier, or cell penetrating peptide [131]. Empirical investigations utilizing animal models have substantiated the therapeutic efficacy of mitochondrial transplantation across a spectrum of disease paradigms, like cardiac ischemia–reperfusion injury, acute respiratory distress syndrome, PD, spinal cord injury, hepatic and renal injury, and sepsis-induced multiple organ dysfunction. Furthermore, a plethora of clinical investigations have delineated the application and effectiveness of mitochondrial transplantation too [119]. The execution of mitochondrial component transplantation not only elucidates molecular mechanisms underpinning tissue revitalization but also presents prospective avenues for future therapeutic interventions [120,132,133].

## 13. Conclusions

The information collected in this review may provide a new insight to better clarify how mitochondrial dysfunction can be detrimental for endothelial cells and induce the onset of various organ disorders and a wide range of diseases, as well as aging. The collected issues put into perspective how important mitochondria are for cell integrity maintenance and especially how they will impact on vascular and general health. Furthermore, from the data collected it demonstrates how mitochondria may turn out to represent a good therapy basis for pathological cases in relation to diseases’ prophylaxis. In addition, innovative methods, such as mitochondrial transplantation and gene therapy, further expand the therapeutic scope toward the treatment of diseases linked to mitochondrial damage. In conclusion, the combination of mitochondrial-targeted therapies with precision medicine advances may provide great promise for personalized approaches in managing complex diseases. However, further research into the function of mitochondria within endothelial cells is critical for a better understanding of physiologic and pathophysiologic aspects and for the design of effective, targeted strategies against the complex processes of aging and chronic diseases.

## Figures and Tables

**Table 1:** Mitochondria and endothelial cell involvement in health and diseases.

Role	Mechanisms	Action/Involvement	References
Endothelium function/dysfunction	Barrier, nutrient transportation, and waste products elimination; mitochondrial dysfunction, swelling, and apoptosis; enhancement of Aβ production; morphological changes in mitochondria; ATP reduction and ROS increase	Maintaining vascular tone and function; cardio-metabolic disease; AD; liver diseases; aging; renal diseases	[1,43,45,62]
Mitochondria in endothelial cells	Oxygen sensors and ROS production; inflammation, redox signaling, AMPK, and PGC pathways; TRPV4 expression and calcium influx; morphology alteration; reduction in mitochondrial function	Aging; cardio-metabolic diseases; neurodegenerative diseases; AD; renal diseases; ALS	[6,7,65,84]
ROS release	NO bioavailability reduction, impaired endothelial dependent vasodilation; Ca^2+^ and eNOS activity regulation; elevated ROS levels	Arterial stiffness; maintaining vascular tone and function; aging	[19,41,46]
Mitochondrial structure/function	Mitochondrial dynamics’ regulation; ETC	Maintaining endothelial cell’s function; ATP production	[26]
Mitochondrial function targeted drugs	Removal of damaged mitochondria; regulation of AKT, nuclear factor erythroid 2-related factor 2, Janus kinase/signal transducer pathways and the activator of transcription, nucleotide-binding and oligomerization domain-like receptor familypyrin domain-containing 3 inflammasome, and MAPK; anti-inflammation and anti-amyloid	Regulation of health and pathological conditions; alleviation of neurodegenerative disorders and cognitive function; mitigating AD, PD, HD, and ALS progression	[10,91,92,93,94,95,96,97]

AD: Alzheimer’s disease, ALS: amyotrophic lateral sclerosis, AKT: protein kinase B, AMPK: AMP-activated protein kinase, ATP: adenosine triphosphate, eNOS: endothelial nitric oxide synthase, ETC: electron transport chain, HD: Huntington’s disease, MAPK: mitogen-activated protein kinase, NO: nitric oxide, PD: Parkinson’s disease, PGC: peroxisome proliferator-activated receptor gamma coactivator, ROS: reactive oxygen species and TRPV4: transient receptor potential vanilloid 4.
